# Chemically and Thermally Crosslinked PVA-Based Membranes: Effect on Swelling and Transport Behavior

**DOI:** 10.3390/polym11111799

**Published:** 2019-11-01

**Authors:** Edyta Rynkowska, Kateryna Fatyeyeva, Stéphane Marais, Joanna Kujawa, Wojciech Kujawski

**Affiliations:** 1 Normandie Université, UNIROUEN, INSA Rouen, CNRS, Polymères Biopolymères Surfaces (PBS), 76000 Rouen, France; edyta.rynkowska@wp.pl (E.R.); stephane.marais@uni-rouen.fr (S.M.); 2 Faculty of Chemistry, Nicolaus Copernicus University in Toruń, 7, Gagarina Street, 87-100 Toruń, Poland; joanna.kujawa@umk.pl

**Keywords:** poly(vinyl alcohol), sulfosuccinic acid, chemical and thermal crosslinking, water permeation, contact angle measurements

## Abstract

The novel poly(vinyl alcohol) (PVA)-based membranes were prepared using the two-step crosslinking approach: the chemical crosslinking of PVA using sulfosuccinic acid (SSA) (0–50 wt.%) and the thermal treatment (120–160 °C). The membrane composition and crosslinking temperature were optimized in terms of the mechanical and transport properties. The FTIR-ATR analysis revealed that the increase of the SSA concentration and crosslinking temperature resulted in the rise of the ester bond bands intensity due to the esterification reaction between PVA and SSA. As a consequence, the PVA-based membrane with 50 wt % SSA and crosslinked at 140 °C showed the reduced Young’s modulus (from 1266.2 MPa to 1.4 MPa) and elongation at break (from 316% to 66%) in comparison with the pure PVA membrane. The studied swelling behavior of the obtained membranes revealed significantly higher water sorption than that in methanol and propal-2-ol whatever the crosslinking temperature. The performed studies provide a new way of tailoring the membrane physicochemical properties, in particular, the surface hydrophilicity. In addition, the obtained results are crucial for the design and elaboration of the polymer membranes for the pervaporative separation of the liquid-liquid mixtures, in particular, for the alcohol dehydration.

## 1. Introduction

Poly(vinyl alcohol) (PVA) is a semi-crystalline water-soluble synthetic polymer widely used in industry due to its multiple features including non-toxicity, biocompatibility, and biodegradability properties, as well as its high barrier properties towards the oxygen and aroma [1,2,3,4]. In addition, it has good film/gel-forming, adhesive, and emulsifying properties [3,5,6]. PVA is a highly hydrophilic polymer; therefore, its properties are strongly influenced by the humidity level. Moreover, PVA swells or even can be dissolved during the contact with liquid water which is its main drawback [6]. In order to reduce the water solubility of the PVA-based materials the chemical and/or thermal crosslinking of the hydroxyl groups of PVA can be performed. In addition, the PVA crosslinking allows improving the mechanical properties, thermal stability, and the separation ability of the material [1,4,7,8,9]. Different crosslinking agents have been already used, such as glutaraldehyde (GA) [10,11,12,13], glyoxal [14], maleic acid [15], citric acid [16], trisodium trimetaphosphate (STMP) [17], sodium hexametaphosphate (SHMP) [17], dianhydrides [18], succinic acid (SA) [19], and sulfosuccinic acid (SSA) [19,20].

The crosslinking of the PVA-based membranes with GA resulted in a significant decrease of the water solubility (from 59% to 40% for pure PVA and PVA with 4% GA, respectively), the water swelling (from 350% to 250%), and the water uptake (from 53% to 38%), which confirmed the improvement of the membrane resistance towards water [4]. It was indicated that the increase of the crosslinking degree led to the reduction of the amount of the water molecules passing through the PVA membranes due to the decrease of the free volume space in the membrane structure [1,2,4]. The crosslinking with poly(ethylene glycol) (PEG) diacylchloride ensured the insolubility of PVA in water by affecting its crystalline structure and causing lower porous volume than that obtained during the reaction with GA [8].

It was reported that the various amount of GA (0–100 μL) used for the PVA-membranes crosslinking can improve the anion selectivity (45–261) in the process of base recovery (in the range of 0.00255–0.0107 m·h^−1^) from industrial wastewater [13]. Liu et al. [12] reported the use of the PVA membranes crosslinked with GA and annealed at 130 °C for the ethanol dehydration by pervaporation. It was found that decrease of the ethanol concentration in the feed from 90 to 80 wt.% increased the water permeance reflected by the increase of water flux from ca. 140 to 450 g·m^−2^·h^−1^ [12]. The PVA crosslinked with dianhydrides (3,3′,4,4′-benzophenone tetracarboxylic dianhydride (BTDA), 4,4′-oxydiphthalic anhydride (ODPA), and pyromellitic dianhydride (PMDA)) revealed the typical trade-off in the ethanol dehydration by pervaporation, namely the PVA crosslinking with dianhydrides resulted in the increase of fluxes (from 120.2 to 190.8 g·m^−2^·h^−1^) and in the decrease of separation factor (from 306 to 58) [18].

The use of trisodium trimetaphosphate (STMP) and sodium hexametaphosphate (SHMP) as crosslinkers for PVA revealed that the pH value had a primary influence on the PVA crosslinking density [17]. It was noted that the crosslinking with STMP at pH 10 resulted in the formation of the triphosphate arms.

In addition to the water resistance, the thermal behavior of crosslinked PVA films also changed as the effect of the modification [4]. It was shown that the crosslinker type and the conditions of the membrane preparation had a strong influence on the PVA structure. For example, the crystallinity of PVA increased after the crosslinking using GA in the ethanol solution, which was confirmed by the increase of the 2θ peak intensity at 20° and by the increase of the melting enthalpy (*ΔH_m_*) from 39 J·g^−1^ to 41 J·g^−1^ as compared with the pure PVA membrane [10]. The crosslinking with GA of the PVA-based membranes containing poly(acrylic acid) (PAA) also influenced the membrane transport and separation properties during the pervaporative separation of the dimethyl carbonate/methanol mixture [21]. The increase of the GA content from 3 to 9 mol.% per mole of the PVA repeating unit enhanced the affinity of the PVA-PAA membranes to methanol. This fact was related to the increase of the crosslinking degree as well as to the amount of the unreacted aldehyde groups. Also, the gradual increase of the methanol flux from 154 g·m^−2^·h^−1^ to 366 g·m^−2^·h^−1^ was observed with the GA content increasing from 3 mol.% to 9 mol.%. The maximum separation factor equal to 37 was obtained for the membrane with 6 mol.% GA [21].

Other approaches were also studied to improve the crosslinking efficiency of the PVA membranes. Kumeta et al. investigated the influence of the heat-treatment conditions and the effect of the neutralization degree for the PVA-based membranes crosslinked with PAA taking into account that the PVA: PAA weight blend ratio equals 8:2 [22]. It was pointed out that the use of the non-neutralized PAA resulted in the drop of the crosslinking performance. This result was related to the increased number of the hydroxyl groups that underwent the oxidation reaction instead of the esterification reaction [22]. A significant difference in the mechanical properties of the membranes chemically crosslinked with GA and heat-treated porous PVA membranes was also reported [23]. The tensile strength of the PVA membranes crosslinked with 3 wt.% GA during 5–30 min was enhanced with the increase of the crosslinking degree. In addition, the tensile strength and strain at break of the crosslinked membranes were found to be higher and lower, respectively, compared to those of the membrane heated at 120 °C during 1–3 h with 3 wt.% GA. The chemically crosslinked PVA membranes studied in the wet state showed the decrease of the tensile strength and strain at break. When a polymer was solvated with water molecules, the polymer chains were extended. However, their expansion was restricted by the presence of crosslinking bonds, which contributed to the increasing of the inner stress with increasing a crosslinking degree during the swelling process. On the contrary, the heat-treated membranes in the wet state possessed superior mechanical properties in comparison with those in the dry state [23]. For example, strain at break of the heat-treated membranes in the wet state was around twice higher than that for the membranes in the dry state. Furthermore, it was shown that the thermal treatment of the PVA-based membranes combined with the chemical crosslinking led to the enhancement of the crosslinking efficiency and water resistance of the PVA-based membranes [22,23]. Thus, it appears to be a promising approach to the improvement of the membrane performance in separation processes.

The aim of this work was to carry out the chemical crosslinking using SSA followed by the thermal crosslinking of the PVA-based membranes in order to improve the PVA separation behavior during the alcohol dehydration. For this purpose, the influence of the SSA concentration and thermal treatment conditions was evaluated by the swelling measurements in the liquids of different polarity (water, methanol and propan-2-ol) as well as by the water vapor permeation kinetics. This work also allows getting a deeper insight into membrane modifications after the chemical and thermal crosslinking reaction, in particular concerning the membrane wettability and surface free energy.

## 2. Experimental

### 2.1. Materials

PVA powder (Elvanol 71-30, fully hydrolyzed, molecular weight of 100,000 Da) was kindly provided by DuPont (USA). SSA (70 wt.% solution in water) was purchased from Merck. Methanol and propan-2-ol as well as glycerol and diiodomethane were delivered by Fischer Chemical. Solvents of the analytical reagent grade and the ultrapure water deionized by Milli-Q (18.2 MΩ·cm, Millipore^®^) were used.

### 2.2. Membrane Preparation

The aqueous 10 wt.% PVA solution was prepared by dissolving the PVA powder in deionized water by stirring under the reflux at 100 °C for at least 6 h. After cooling to room temperature, a certain amount of SSA (from 5 up to 50 wt.%) was added to the solution. The solution was vigorous stirred at the room temperature (21 ± 3 °C) during 24 h. The solution was degassed and cast on a hydrophobized glass plate using the film applicator (Doctor Blade^®^). The cast polymer films were dried in the oven at 30 °C for at least 24 h. The dried membranes were stored in a desiccator over P_2_O_5_ and treated as follows: (1) 1 h at 25 °C, (2) 6 h at 80 °C, (3) 1 h at a given crosslinking temperature (120 °C, 140 °C or 160 °C) under the vacuum. The thickness of the prepared membranes was 180 ± 10 µm.

### 2.3. Membrane Characterization

The Fourier transform infrared spectroscopy with attenuated total reflectance (FTIR-ATR) (equipped with Ge crystal) analysis was performed using the Nicolet FT-IR apparatus (ThermoFischer, Avatar 360 Omnic Sampler) in the range of 4000–500 cm^−1^ with resolution of 4 cm^−1^ and 256 scans. The transmittance values were normalized according to the invariable -CH_2_- stretching vibration band of the PVA backbone (ν = 2939 cm^−1^).

The mechanical properties of the crosslinked PVA membranes were evaluated using the Instron 5543 machine. Tensile deformation was determined at a cross-head speed of 1 mm·min^−1^ for the samples with length of 30 mm and width of 5 mm. The tests were carried out at ~23 °C and ~50% relative humidity (RH). Five samples were tested for each membrane.

The thermal properties of membranes were studied using the thermogravimetric analysis (TGA) Q 500 (TA Instruments, New Castle, DE, USA). The thermogravimetric analysis was carried out at the temperature from 25 °C to 600 °C with the heating rate equal to 10 °C·min^−1^ under the nitrogen atmosphere (10 mL·min^−1^).

The morphology of the prepared membranes based on poly(vinyl alcohol) and sulfosuccinic acid (PVA-SSA) was studied by the scanning electron microscopy (SEM) using the SEM 1430 VP microscope (LEO Electron Microscopy Ltd., Macclesfield, UK). Prior to the analysis, the membranes were frozen in liquid nitrogen for several minutes and broken to obtain the cross-section images. The membrane samples were sputtered with gold. The SEM images were obtained at 2 kV.

The surface free energy (*SFE*) of the PVA-SSA membranes was determined at ~22 °C and 50% RH by the contact angle measurements using water, glycerol and diiodomethane. The contact angle was measured with accuracy of ±3° using the Multiscope apparatus (Optel, Germany) and applying the sessile drop method. The contact angles of each drop of a given liquid were measured after 5 s just after the drop deposition on the membrane surface. The surface free energy (*γ*) was calculated by means of the least-squares fit applying the Owens-Wendt method [24].

### 2.4. Swelling Measurements

The swelling behavior of the PVA-SSA membranes in a liquid solvent (water, methanol and propan-2-ol) was studied at 25 ± 1 °C. The membrane swelling was evaluated gravimetrically on the basis of the membrane solvent uptake estimated from the mass gain between the dried membrane and the membrane equilibrated in a given solvent. Prior to the experiment, the membrane was dried in the desiccator under vacuum at ambient temperature for more than 48 h up to a constant membrane weight (*m_dry_*). Subsequently, the membrane was immersed into the solvent. A sample was taken out from the solvent, the excess of solvent from the membrane surface was wiped with tissue, and the sample was immediately weighted (*m_wet_*). This procedure was repeated until the equilibrium state was reached. The mass swelling degree (*SD_m_*) and molar swelling degree (*SD_M_*) of the equilibrated membrane were calculated according to the following equations [25]:

(1) $$SD_{m}=\frac{m_{wet}-m_{dry}}{m_{dry}}\;(g\;\mathrm{solvent}/g\;\mathrm{dry}\;\mathrm{membrane})$$

(2) $$SD_{M}=\frac{SD_{m}}{M_{sol}}\;(\mathrm{mol}\;\mathrm{solvent}/g\;\mathrm{dry}\;\mathrm{membrane})$$

where *m_wet_* and *m_dry_* are the weights of the dry and solvent-equilibrated PVA-SSA membrane, respectively; *M_sol_* is the solvent molecular mass.

### 2.5. Water Vapor Permeation Measurements

The permeation measurements were carried out using the permeameter described in details elsewhere [26,27]. Prior to the measurement, a membrane sample was mounted in the permeation cell and dried by circulating dry nitrogen on both sides of the membrane till the lowest dew point temperature was reached, which corresponds to the lowest amount of humidity in the system. Subsequently, the stream of water vapor at the desired RH was introduced into the upstream compartment while the downstream compartment was continuously swept out by the stream of dry nitrogen. Due to the driving force created by the gradient in water activity on both sides of the membrane, the water molecules permeated through the membrane. The water concentration in the downstream compartment collected by the sweeping gas was monitored using a chilled mirror hygrometer (General Eastern Instruments, USA) connected to the data acquisition system. The data were recorded until the stationary state in the system was reached. In this study, the permeation experiments were carried out in a thermostatic chamber Binder KT-115 at 25 ± 1 °C applying various RH values (24–91% RH).

The permeability coefficient *P* expressed in Barrer units (1 Barrer = 10^−10^ cm^3^_STP_·cm·cm^−2^·s^−1^·cm Hg^−1^) was determined from the stationary flux *J_st_* according to the following equation:

(3) $$P=\frac{J_{st}L}{\Delta p}$$

where *L* is the membrane thickness and Δ*p* is the pressure difference on both sides of the membrane. For comparison with literature data, the permeability coefficient was also expressed in g·mm·m^−2^·kPa^−1^·h^−1^ and g·m·m^−2^·atm^−1^·d^−1^.

## 3. Results and Discussion

### 3.1. FTIR-ATR Analysis

**Figure 1:**
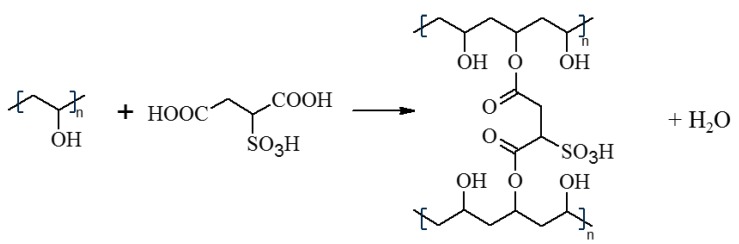
Scheme of the crosslinking reaction between poly(vinyl alcohol) (PVA) and sulfosuccinic acid (SSA).

**Figure 2:**
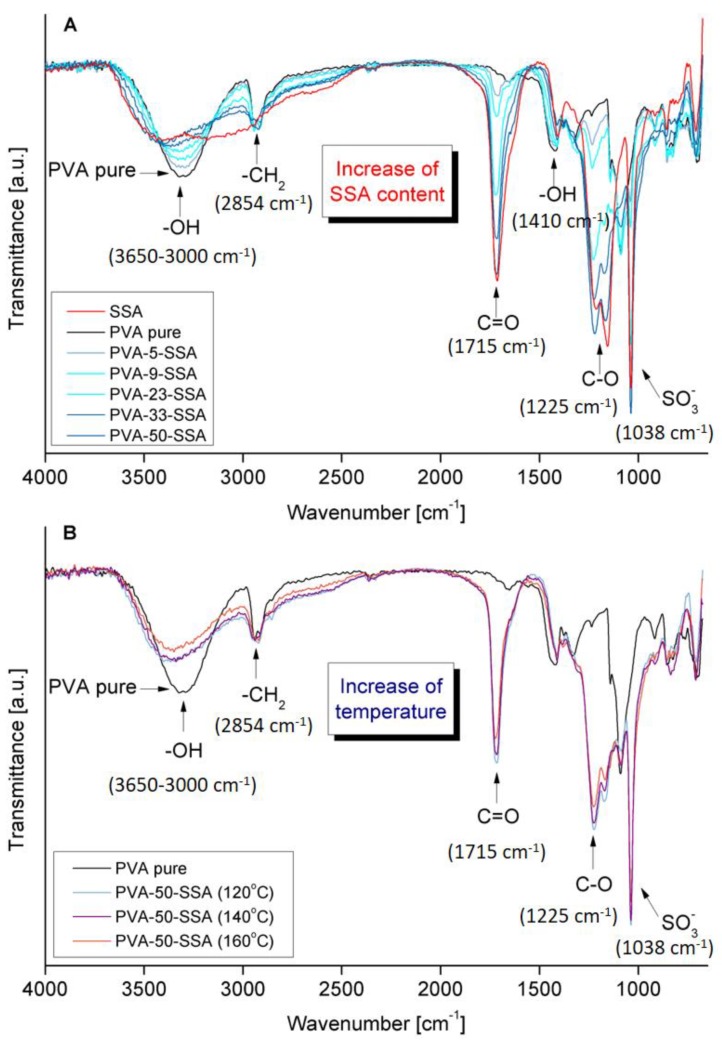
FTIR-ATR spectra of the crosslinked PVA-based membranes: (**A**) heat-treated at 120 °C as a function of the SSA content (5–50 wt.%) and (**B**) containing 50 wt.% SSA as a function of the crosslinking temperature (120–160 °C).

In order to reveal the efficiency of the crosslinking reaction between PVA and SSA (Figure 1), the FTIR-ATR analysis was applied. The FTIR-ATR spectra of the pure and crosslinked PVA membranes are given in Figure 2. The pristine PVA membrane presents a characteristic broad and large O–H stretching band (3685–3010 cm^−1^); symmetric (2929–2915 cm^−1^) and asymmetric (2854 cm^−1^) –CH_2_– bands; as well as a C–O stretching band (1238 cm^−1^) [1,28]. The FTIR-ATR spectrum of the SSA solution (Figure 2A) possesses the band of O–H stretching vibrations of the water molecules and the carboxylic acid groups (–COOH) in the range from 3675 to 3000 cm^−1^. The stretching vibration band of SO_3_^−^ groups is found at 1038 cm^−1^. The presence of the sulfonic groups in the FTIR-ATR spectrum is due to the dissociation of –SO_3_H in the SSA molecules [1]. The bending band of the water molecules can be found in the region around 1640 cm^−1^ which confirms the high water content in the SSA solution; however, this band is overlapped with the C=O stretching band at 1714 cm^−1^ from the –COOH group (Figure 2).

The spectra of the PVA-SSA membranes crosslinked using SSA of various concentrations and heated at different crosslinking temperature are shown in Figure 2. The obtained spectra of the crosslinked PVA-SSA membranes showed significant differences compared to the FTIR spectrum of the pure PVA membrane revealing the influence of the SSA addition (Figure 2A). The increasing content of SSA in the PVA-based membranes at a given crosslinking temperature resulted in the increase of C=O and C–O bands intensity of –COO– ester groups [29]. The C=O band was found at around 1718 cm^−1^ whatever the SSA concentration, whereas the C–O band observed at 1240 cm^−1^ for pure PVA was shifted to lower wavenumbers with the increasing SSA content (up to 1217 cm^−1^ for the PVA-SSA membrane containing 50 wt.% SSA). Moreover, the intensity of the O–H group bond was reduced with the increasing SSA content, which is related to the crosslinking reaction. The obtained result proves the PVA crosslinking by the esterification reaction between O–H groups in PVA and –COOH groups in SSA (Figure 1) [2,28,29]. The increasing content of SSA increases the intensity of the symmetric stretching vibration band of -SO_3_^−^ group in the region at ~1040 cm^−1^ (Figure 2A).

The remarkable influence of the crosslinking temperature (120 °C, 140 °C, and 160 °C) on the spectral behavior of the PVA-SSA membranes is clearly shown in Figure 2B. It can be seen that the raise of the curing temperature from 120 °C to 160 °C results in the decrease of the O–H group bands intensity. Simultaneously, the intensity of the C=O and C–O symmetric stretching vibration bands increases.

The works of Martínez-Felipe et al. [1], Rhim et al. [2], and Kudoh et al. [19] reported the importance of the thermal treatment for the PVA-based membranes reinforced with SSA as a crosslinking agent. The influence of the crosslinker on the membrane properties was studied in terms of their application as the polyelectrolyte membranes. Martínez-Felipe et al. indicated that the esterification crosslinking reaction occurred in all prepared membranes containing 5–30 wt.% SSA [1]. The additional membrane heating at 110 °C enhanced the thermal stability and the crosslinking of membranes was revealed by the decrease of the bands intensity of –OH groups in the 3600–3000 cm^−1^ region and by the increase of the intensity of C=O and SO_3_^−^ bands. Therefore, the presence of SSA in the membrane increased the proton conductivity as the increase of the ion-exchange capacity from 0.052 meq·g^−1^ to 0.141 meq·g^−1^ for membranes containing 5 wt.% and 30 wt.% SSA, respectively, was observed [1].

Kudoh et al. crosslinked PVA with SA and SSA [19]. It was shown that the crosslinking efficiency increased with increasing the SA content and the crosslinking temperature. In the case of SSA, the crosslinking reaction was effective at the low SSA content (5–10 wt.%) in the whole investigated crosslinking temperature range from 140 °C to 200 °C, which was confirmed by the low dissolution rate of the PVA-SSA membranes. However, the dissolution rate of the membranes containing 20 wt.% SSA was nearly two times higher than that of the PVA pure membrane after heating at 180 °C and 200 °C. It was pointed out that SSA was used in excess; therefore, the unreacted SSA molecules were dissolved while swelled in hot water. Nevertheless, it was observed that SSA was more reactive than SA in the crosslinking by the esterification reaction even if the increase of a SA content and the temperature of crosslinking decreased the dissolution rate. For example, the PVA-SSA membranes containing 5 wt.% or 10 wt.% SSA had the dissolution rate not exceeding 10%, whereas the membranes crosslinked with 6 wt.% SA and heated at 140 °C exhibited the dissolution rate around 55%.

### 3.2. Thermal Properties

**Figure 3:**
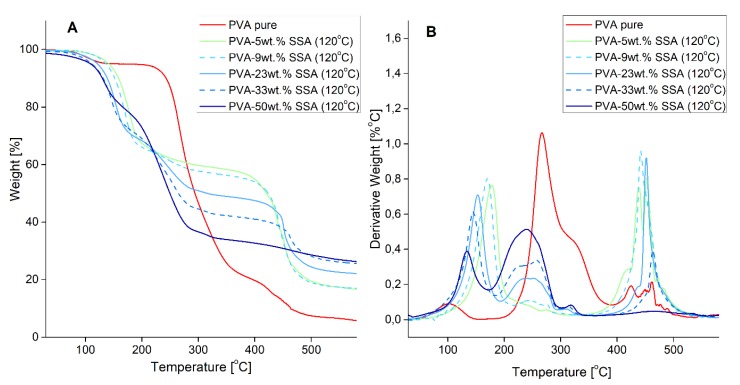
Thermogravimetric analysis of the heat-treated at 120 °C PVA-based membranes containing 5–50 wt.% SSA: (**A**) thermogravimetric curves and (**B**) differential thermogravimetric curves.

The thermal stability of the pristine PVA membrane and crosslinked PVA-SSA membranes heat-treated at 120 °C for 1 h was analyzed using the TGA analysis. TGA and derivative thermogravimetric (DTG) curves are presented in Figure 3. The thermal degradation of pristine PVA follows three main degradation steps. The first stage includes the loss of water molecules trapped in the hydrophilic PVA and is characterized by the peak at around 100 °C. The second intensive peak in the range of 200–380 °C can occur due to the loss of –OH groups and the deacetylation of PVA chains [30]. The third degradation step observed at 400–500 °C is assigned to the degradation of the PVA backbone [1,11].

In the case of the PVA-SSA membranes, three main degradation steps can be also distinguished. The first strong weight loss observed from around 100–200 °C can be attributed to the loss of water molecules strongly bounded to the polymer structure. The peak at 200–300 °C corresponds to the desulfonation as well as to the loss of -OH and acetate groups from the polymer structure related with the break of ester bonds created between PVA and SSA (Figure 1). The weight loss in this range increases with the increasing concentration of a crosslinking agent [31]. Moreover, this peak is shifted to a lower temperature for the crosslinked membranes in comparison with the pure PVA membrane (Figure 3). The degradation of the PVA-SSA main chains occurs in the region of 400–500 °C [1,2] and is more pronounced than in the case of pure PVA (Figure 3).

It was noticed that the increase of the crosslinking temperature from 120 °C to 160 °C led to a slight increase of the degradation temperature at a given weight loss (Table 1), thus indicating the improvement of the thermal stability of the elaborated PVA-SSA membranes. Indeed, for the PVA-SSA membrane containing 33 wt.% SSA and annealed at 120 °C and 160 °C, the 5% weight loss is observed at 111 °C and 127 °C, respectively.

### 3.3. Mechanical Properties

**Figure 4:**
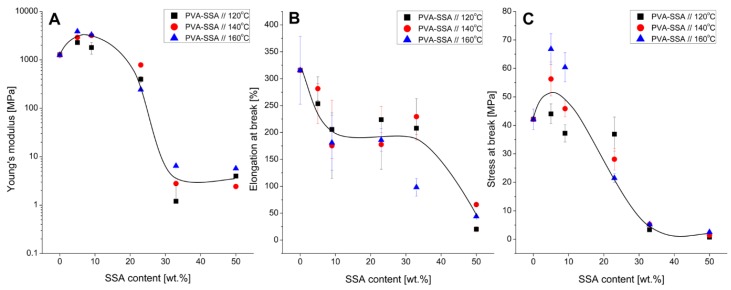
Influence of the crosslinking temperature on the mechanical properties of the PVA-SSA membranes: (**A**) Young’s modulus, (**B**) elongation at break, and (**C**) stress at break. The solid lines are only eye guided.

The influence of the crosslinking agent content and the crosslinking temperature on the tensile behavior of the PVA-SSA membranes is shown in Figure 4. The mechanical properties of the elaborated membranes were evaluated in terms of the Young’s modulus (*E*) (Figure 4A), elongation at break (*ε_max_*) (Figure 4B), and stress at break (*σ_max_*) (Figure 4C). The Young’s modulus known as an elastic modulus reflects the material stiffness towards deformation under applied loading, and it is calculated from the slope of the straight-line part of the stress-strain curve in the elastic deformation region. The elongation and stress at break are the elongation (*ε_max_*) and maximum tensile stress (*σ_max_*) values required by the tested sample during the tensile test. In general, high values of *E*, *ε_max_* and *σ_max_* display the hard and tough behavior of the material, while their low values characterize the soft and weak behavior [3].

The Young’s modulus, stress at break, and elongation at break values for the pristine PVA membrane are *E* = 1266 ± 168 MPa, *σ_max_* = 42 ± 4 MPa, and *ε_max_* = 315 ± 63%, respectively. The increase of the SSA content up to 9 wt.% for the membranes crosslinked at 140 °C resulted in the increase of the Young’s modulus up to 3196 ± 338 MPa and the stress at break up to 46 ± 3 MPa compared to the pristine PVA membrane. The simultaneous decrease of the elongation at break value (176 ± 61%) is related to the enhanced membrane stiffness and the reduced polymer chains’ flexibility [3,16,32]. The further raise of a SSA content resulted in the drop of the Young’s modulus (Figure 4A) and the stress at break (Figure 4C), whereas the elongation at break remained at the constant level of about 200% followed by the sharp decrease to ~50% for the membrane containing 50 wt.% SSA (Figure 4B). The PVA-SSA membrane containing 50 wt.% SSA exhibits a brittle behavior, whatever the crosslinking temperature, as shown by the significant decrease of the elongation at break and stress at break values—*ε_max_* = 66 ± 2.9% and *σ_max_* = 1.4 ± 0.1 MPa. This can be explained by the high crosslinking density, which hinders the plastic deformation of the studied membrane [16]. In the same way, Dadfar et al. pointed that the increase of the GA content from 3 wt.% to 9 wt.% increased the tensile strength (from 8 MPa to 13 MPa) and decreased the elongation at break (from 270% to 200%) [4]. Vashisth et al. indicated the improved tensile strength of the PVA-gellan nanofibers due to the application of the two-step crosslinking involving the heat treatment and exposure to methanol and CaCl_2_ [33]. The similar behavior can be noticed for the heat-treated PVA-SSA membranes at a low content of SSA reflected by the increased stress at break values with increasing the crosslinking temperature (Figure 4C).

In general, the hydrogen bonds and hydrophobic interactions stabilize the pristine PVA membrane. The crosslinking of PVA with SSA results in the creation of covalent bonds between the PVA chains and crosslinking agent (Figure 1), which leads to the increase of the stress at break [4] (Figure 4C). However, the increase of the SSA concentration over 10 wt.% causes the progressive deterioration of the mechanical properties as observed by the drop of the stress at break, elongation at break, and Young’s modulus values (Figure 4) due to the exceeded inner stress in the PVA-SSA membranes [7,23]. It is known that in the case of the excessive crosslinking the internal stresses occur and deteriorate the mechanical properties of polymer membranes [34,35,36]. This phenomenon is widely studied in the case of the epoxy polymers and acrylate networks showing that the internal stress is extended through the membrane thickness from the surface to the center [36]. Such behavior was also observed for the PVA-based membranes crosslinked with GA [34]. It was shown that the increase of the GA content from 16 wt.% to 25 wt.% decreases the flexural strength and modulus. Korsmeyer et al. noticed that the crosslinking with a high GA content and subsequent drying of the PVA-based gels can create the microvoids and cracks due to the pronounced inner stress inside the highly crosslinked polymer networks [35]. Rey et al. pointed out the significance of the inner stress after annealing and post-annealing treatment for the dimethacrylate/styrene films [36]. The value of the internal stress was associated with the double bond conversion and crosslinking density.

The degree of the PVA crosslinking depends on both the chemical and thermal crosslinking. Riyajan et al. reported that the increase of the maleic acid content (from 10 wt.% to 60 wt.%) and the time of crosslinking (from 10 min to 60 min at 120 °C) enhanced the crosslinking degree of the PVA-based membranes [7]. For example, the increasing content of maleic acid (from 10 wt.% to 60 wt.%) improved the tensile strength (from 12 MPa to 32 MPa) of the studied PVA-based membranes and this effect was more pronounced when the membrane was heated at 120 °C.

Birck et al. discovered that the PVA-based membranes crosslinked with 10 wt.% and 40 wt.% of citric acid (CTR) required heating for 40 min and 120 min at 130 °C, respectively, in order to reach the complete crosslinking reaction between PVA and CTR [16]. The tensile measurements testified that the studied PVA-CTR membranes containing 40 wt.% CTR and heated at 130 °C during 40 min were brittle because of the high crosslinking density hindering the plastic deformation, reflected by the nominal stress and nominal strain equal to around 40 MPa and 0.01, respectively. In the case of the PVA-based membranes crosslinked with 10 wt.% CTR, it was shown that the increase of the annealing time up to 360 min decreased the nominal strain from around 2 to 0.3 and increased the nominal stress from around 18 MPa to 23 MPa, as compared with the non-heated PVA-CTR membrane.

### 3.4. Membrane Morphology

**Figure 5:**
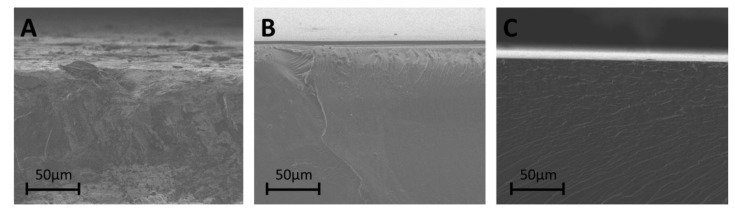
SEM cross-section micrographs of: (**A**) pristine PVA membrane, (**B**) PVA-SSA membrane containing 23 wt.% SSA heated at 140 °C, and (**C**) PVA-SSA membrane containing 33 wt.% SSA heated at 140 °C.

The morphology of the pristine PVA membrane and the heat-treated PVA-SSA membranes was investigated using the SEM analysis. The SEM micrographs of the cross-section view of the pure PVA and PVA-based membranes containing 23 wt.% and 33 wt.% SSA heated at 140 °C are presented in Figure 5. The cross-section of the native PVA membrane is uniform and smooth (Figure 5A). The introduction of 23 wt.% and 33 wt.% of a crosslinking agent (SSA) into the PVA membrane matrix did not change the membrane homogeneity (Figure 5B,C, respectively). In addition, the cross-section SEM micrographs of the elaborated membranes indicated that the obtained membranes are dense and non-porous.

The compatibility of the polymer matrix and the crosslinking agent is a key factor for further possible incorporation of the chosen additives into the crosslinked membrane structure. Indeed, Riyajan et al. reported that the crosslinking PVA-based membranes containing maleic acid enhanced the compatibility of PVA and the used epoxised natural rubber (ENR) [7]. The pristine PVA-ENR blend revealed numerous microphase separations highlighted by the SEM analysis. The crosslinking of PVA with maleic acid resulted in the significant increase of the compatibility of PVA and introduced ENR.

### 3.5. Contact Angle Measurements

In order to control the membrane surface properties, which can be changed by the chemical and physical modification, the direct (e.g., contact angle measurements, drop shape analysis) and indirect (e.g., the Owens-Wendt method for the determination of the membrane total surface free energy) methods can be used. A hydrophilic/hydrophobic character of the membrane can be characterized by measuring the water contact angle determined by aligning a tangent between the surface of the sessile drop and the contact outline with the surface. The drop angle value indicates the affinity between the membrane and water, namely, the decreasing contact angle reflects a higher compatibility of the water molecules and membrane due to the presence of polar groups. In general, the contact angle values much lower than 90° are characteristic of the hydrophilic surface [30,37,38]:

(4) $$\gamma_{lv}\cos\theta =\gamma_{sv}-\gamma_{sl},$$

where *γ_lv_* is liquid/vapor interfacial tension (i.e., liquid surface tension), θ is the contact angle, *γ_sv_* is the solid free energy, and *γ_sl_* is the solid/liquid interfacial free energy. The membrane surface properties can be also characterized by the total surface free energy (*SFE*) value, i.e., a surface tension *γ* determined using the contact angle values for two or three solvents. The *SFE* value provides the information about the surface physicochemistry as well as the evaluation of the membrane ability for adsorption, wettability, and adhesion [39].

In this study the *SFE* values were calculated by means of the least-squares fit using the Owens-Wendt approach [38,40,41,42,43]. This approach allows to separate the interfacial tension according to the underlying polar and dispersive interactions between the materials. Thus, the total *SFE* value of the solid is the sum of the two parts (i.e., polar and dispersive interactions). The polar interactions arise from the permanent dipole-permanent dipole interactions or Keesom forces [40,41,42]. They are strong and exist only in polar molecules. The dispersive component (also known as London forces) are weak and arise due to the random fluctuations in the electron density and, hence, lead to induced dipole interactions [40,43]. For the Owens-Wendt method three liquids with known dispersive and polar parts of surface tension are used (namely, water, glycerol and diiodomethane) (Table 2) and, thus, the solid/liquid interfacial free energy and solid surface free energy are determined:

(5) $$\gamma_{sl}=\gamma_{sv}+\gamma_{lv}-2(\sqrt{\gamma_{sv}^{D}\gamma_{lv}^{D}+}\sqrt{\gamma_{sv}^{P}\gamma_{lv}^{P}}$$

where $\gamma_{sv}^{D}$ and $\gamma_{lv}^{D}$ are dispersive components and $\gamma_{sv}^{P}$ and $\gamma_{lv}^{P}$ are polar components of solid and liquid surface energy, respectively.

The obtained results for the heat-treated PVA-SSA membranes revealed that the water contact angle values increased with the SSA concentration increasing (Table 3). This fact means the reduction of the hydrophilic character of the membrane surface due to a lower amount of hydroxyl groups after the esterification reaction with a crosslinking agent (Figure 1), which is in good agreement with the results obtained by the FTIR-ATR analysis (Figure 3). The average water contact angle values for the PVA-based membranes heated at 140 °C and containing 5 wt.% and 33 wt.% SSA were equal to 75° and 89°, respectively (Table 3). The increase of the water contact angles due to crosslinking was also observed for the PVA-gellan nanofibers indicating the improved stability of the PVA-based materials in contact with water [33].

Penkova et al. reported that the increase of the annealing temperature slightly increased the water contact angle testifying to the reduction of the membrane surface hydrophilicity [44]. The PVA-SSA membranes containing 5 wt.% of a crosslinker and heated at 140 °C and 160 °C possess the water contact angle values equal to 75° and 80°, respectively. It was shown that the heat-treatment at 140 °C during 100 min diminished the surface hydrophilic character of the PVA-based materials containing fullerenol as a crosslinker [44]. However, the incorporation of fullerenol revealed the increased hydrophilic character of a film surface compared to the pristine PVA membrane. The water contact angle of the pristine PVA membrane was 66°, whereas the addition of 5% of fullerenol decreased the contact angle value to 45°. Also, the reduction of the hydrophilic character of the membrane surface can be achieved by increasing the UV irradiation dose for the thermoplastic starch-PVA blend films due to the consumption of hydroxyl groups during the crosslinking reaction [32].

Taking into account measurement errors, the *SFE* results indicate that the thermal and chemical crosslinking of the PVA-SSA membranes does not significantly influence both polar *γ^P^* and dispersive *γ^D^* components (Table 3). However, the London dispersive interactions [45] are observed to be predominant as a much higher dispersive component value *γ^D^* is found compared to the polar component *γ^P^* value. It is known that materials with a low surface energy (30–35 mJ·m^−2^) demonstrate great water repellency [30,40,41,42,43,46], which makes them popular for the wettability researches and explains their water resistance.

### 3.6. Water, Methanol and Propan-2-ol Uptake

In order to evaluate the influence of the crosslinking on the swelling behavior of the PVA-based membranes, the membrane swelling measurements were carried out in contact with pure water, methanol, and propan-2-ol. These solvents were chosen taking into account their polarity. According to their relative polarity they can be put in the following order [43]:

water (1) > methanol (0.762) > propan-2-ol (0.546).

The values of the molar swelling degree *SD_M_* of the PVA-SSA membranes annealed at 120 °C, 140 °C and 160 °C in contact with different solvents are calculated according to Equation (2) and gathered in Table 4. It can be seen that the membrane sorption in water is significantly higher than that in methanol and propal-2-ol whatever the crosslinking temperature. For example, *SD_M_* of the PVA-SSA membrane containing 9 wt.% SSA and heated at 140 °C is equal to 362.4 mol solvent/g dry membrane in the case of the water swelling, whereas *SD_M_* for the same membrane swelled in methanol and propan-2-ol is equal to 1.7 mol solvent/g dry membrane and 0.12 mol solvent/g dry membrane, respectively. Thus, a higher membrane swelling degree in water compared to the swelling degree in the solvents of a lower polarity (methanol and propan-2-ol) was noticed.

Besides, the obtained results showed that the membrane swelling degree in water is inversely proportional to the SSA content for all studied membranes except the membrane with 5 wt.% SSA whatever the crosslinking temperature (Table 4). The highest swelling degree in water is observed for the PVA-based membrane containing 9 wt.% SSA, and this value decreases with the increasing content of a crosslinking agent. Such decrease of the water swelling with the increasing crosslinker content is related to the decrease of the polymer network free volume, i.e., the capability of polymer to swell in water progressively decreases [44,45]. It should be also taken into account that the crosslinking agent amount influences the polymer hydrophilic character (Table 3), and, consequently, contributes to the swelling ability of the membrane.

As expected, the alcohol swelling behavior of the PVA membrane is also influenced by the crosslinking reaction (Table 4). The swelling measurements revealed a higher methanol swelling as compared with that in propan-2-ol. This fact is in agreement with the swelling results for the PVA membranes crosslinked with GA obtained by Farid et al. [47]. It was shown that for the PVA-GA membrane the swelling in ethanol is higher than that in propan-2-ol.

The difference in the swelling behavior of the PVA-SSA crosslinked membranes can be explained by the molecular size of a solvent [48]. The size of the studied solvent molecules decreases in the following order:

propan-2-ol (5.12 Å) > methanol (4.10 Å) > water (3.40 Å).

Consequently, it is not surprising that the membrane swelling capacity increases in the same order (Table 4). As it was mentioned before, the crosslinking of the polymer reduces the membrane free volume and, therefore, small solvent molecules can penetrate into the membrane matrix easier and more rapidly. Moreover, as the methanol and propan-2-ol molecules are bigger than the water ones, the penetration of alcohols into the membrane is delayed in comparison with water in the case of the crosslinked membranes.

Penkova et al. indicated the significant influence of the heat-treatment on the crosslinked PVA-fullerenol membranes equilibrated with water and ethanol [44]. The reduced water and ethanol sorption capacity for the PVA membranes annealed at 140 °C for 100 min was found. After the membrane annealing, the water sorption degree decreased from 310% to 87% for the membrane containing 5% of fullerenol. In the case of the ethanol sorption, the heat-treatment decreased the sorption degree from 8.6% to 2.6% for the PVA-based membrane containing 5% of fullerenol [44].

### 3.7. Water Vapor Permeation

The membrane containing 23 wt.% SSA and heat-treated at 140 °C reveals a good mechanical stability (Figure 4). Moreover, it possesses the highest difference of the swelling ability between water and organic solvents (Table 4). Therefore, the behavior of this membrane towards water was investigated by the analysis of the water vapor permeation kinetics. The measurements were carried out at different RH levels in order to study the water transport properties of the crosslinked membrane as a function of the moisture content surrounding the membrane. The water permeability coefficient values obtained for the membrane at RH varied from 24% to 91% are gathered in Table 5. The obtained results indicate that the membrane moisture resistance is strongly influenced by the RH conditions. The increase of the water activity causes the greater water permeation of the investigated membrane and, thus, the increase of the membrane permeability coefficient *P* from 16.6 Barrer at 24% RH to 63865.1 Barrer at 91% RH. Such high dependence of the water permeability on the water activity is in agreement with the results of López-de-Dicastillo et al. [6]. In this case, the PVA/β-cyclodextrin (βCD) composites crosslinked with glyoxal reveal strong dependence on the water vapors presence in the whole investigated RH range (35–70% RH) depending on the crosslinking method. It was stated that the films prepared by the addition of the βCD solution to the previously crosslinked PVA solution (PVA*.CD) and by the crosslinker addition to the aqueous solution containing PVA-βCD ((PVA.CD)*) possessed the highest barrier properties and the best physical properties among tested composites. The asterisk * refers to the crosslinked material. The water permeability coefficient *P* of the pristine PVA membrane was equal to 116.0·10^3^ g·m·m^−2^·day^−1^·atm^−1^ at 70% RH, whereas the *P* values for the PVA*.CD and (PVA.CD)* membranes were much lower—87.5·10^3^ g·m·m^−2^·day^−1^·atm^−1^ and 37.5·10^3^ g·m·m^−2^·day^−1^·atm^−1^, respectively [6]. These results clearly show the improvement of the water barrier properties due to the crosslinking reaction. Moreover, it was observed that for all studied composite membranes the permeability increased with the RH increasing. For example, *P* raised from 2.0·10^3^ g·m·m^−2^·day^−1^·atm^−1^ to 37.5·10^3^ g·m·m^−2^·day^−1^·atm^−1^ for the PVA*.CD membrane with the RH increasing from 35% to 70% RH, respectively.

The improvement of the water barrier properties of PVA by using GA as a crosslinking agent at 25 °C and 60% RH was also reported by Mahdi Dadfar et al. [4]. It was shown that the increasing GA content in the membrane caused the decrease of the water permeability. This result is explained by the fact that the membrane with the highest crosslinker content is characterized by the increased crosslinking degree, which leads to the network density increase and, thus, hinders the transport of the water molecules through the membrane. The water permeability of the membranes decreased from 0.4 g·mm·m^−2^·kPa^−1^·h^−1^ to ~0.15 g·mm·m^−2^·kPa^−1^·h^−1^ for the pristine PVA membrane and the PVA membrane containing 4% GA, respectively.

The water permeability results obtained in the present work seem to be higher than those presented by other researchers due to the difference in the structure of a used crosslinking agent. In any case, the obtained results clearly testify to the improved barrier performance of the PVA membranes after the crosslinking reaction.

## 4. Conclusions

The PVA-based membranes were crosslinked using a two-step approach. The crosslinking effect due to the esterification reaction and the following heat-treatment was evidenced by the FTIR-ATR analysis through the intensity increase of the ester bond bands between PVA and SSA and the decrease of the -OH groups intensity. As a result of the SSA content increase (up to 50 wt.%), the crosslinking density raised significantly, which was confirmed by the decrease of the values of Young’s modulus, stress at break, and elongation at break whatever the applied crosslinking temperature (120–160 °C). Moreover, the increase of the water contact angles by 15° and 7° with the SSA concentration and the annealing temperature increasing, respectively, was noted, thus confirming the hydrophilic character of the PVA-SSA membrane surface. The swelling studies of the PVA-SSA membranes in contact with pure water, methanol and propan-2-ol revealed that the membrane swelling decreased with the increase of the SSA concentration for all studied crosslinking temperatures. Besides, the highest swelling was observed for the membranes equilibrated with pure water. The elaborated membranes are characterized by a very low swelling capacity for the organic solvent in comparison with water, which indicates that the PVA membranes crosslinked with SSA possess a high potential for the pervaporative separation processes with the purpose of the alcohol dehydration. Therefore, the permeability measurements of pure methanol and propan-2-ol as well as of alcohol solutions with different water concentration will be performed. In addition, the aging study of the elaborated membranes under different temperature conditions will be the subject of further research.

## Figures and Tables

**Table 1:** Characteristic degradation temperature of the studied PVA-SSA membranes.

Membrane	Degradation Temperature [°C]	
	at 5% Weight Loss	at 10% Weight Loss
PVA pure	163	243
PVA-5 wt.% SSA (120 °C)	142	159
PVA-5 wt.% SSA (140 °C)	143	159
PVA-5 wt.% SSA (160 °C)	144	158
PVA-9 wt.% SSA (120 °C)	137	152
PVA-9 wt.% SSA (140 °C)	134	150
PVA-9 wt.% SSA (160 °C)	145	159
PVA-23 wt.% SSA (120 °C)	121	139
PVA-23 wt.% SSA (140 °C)	125	140
PVA-23 wt.% SSA (160 °C)	126	143
PVA-33 wt.% SSA (120 °C)	111	132
PVA-33 wt.% SSA (140 °C)	120	137
PVA-33 wt.% SSA (160 °C)	127	142
PVA-50 wt.% SSA (120 °C)	109	130
PVA-50 wt.% SSA (140 °C)	120	136
PVA-50 wt.% SSA (160 °C)	118	134

**Table 2:** Dispersive *γ^D^* and polar *γ^P^* components of water, glycerol, and diiodomethane [38,39].

Liquid	*γ*	*γ^D^*	*γ^P^*
	mN·m^−1^	mN·m^−1^	mN·m^−1^
Water	72.8	21.8	51.0
Glycerol	63.4	37.0	26.4
Diiodomethane	50.8	50.8	0.0

**Table 3:** Water contact angle and surface free energy dispersive *γ^D^* and polar *γ^P^* components for the crosslinked PVA-SSA membranes.

Membrane	Crosslinking Temperature [°C]	Water Contact Angle [°]	*γ^D^* [mJ·m^−2^]	*γ^P^* [mJ·m^−2^]	*SFE* [mJ·m^−2^]
PVA-5 wt.% SSA	120	86	33.2	1.3	34.5
PVA-9 wt.% SSA		91	34.7	0.8	35.5
PVA-23 wt.% SSA		96	29.4	0.7	30.1
PVA-33 wt.% SSA		92	31.0	0.8	31.8
PVA-5 wt.% SSA	140	75	33.1	2.8	35.9
PVA-9 wt.% SSA		84	32.6	1.5	34.1
PVA-23 wt.% SSA		98	31.2	0.3	31.5
PVA-33 wt.% SSA		89	29.2	1.3	30.5
PVA-5 wt.% SSA	160	80	33.6	1.8	35.4
PVA-9 wt.% SSA		89	33.5	0.9	34.3
PVA-23 wt.% SSA		87	29.0	2.3	31.3
PVA-33 wt.% SSA		91	30.5	2.7	33.1

**Table 4:** The molar swelling degree *SD_M_* of the heat-treated PVA-SSA membranes in water, methanol and propan-2-ol as a function of the SSA concentration and crosslinking temperature.

Membrane	Crosslinking Temperature [°C]	*SD_M_* [mol Solvent/g Dry Membrane]		
		Water	Methanol	Propan-2-ol
PVA-5 wt.% SSA	120	179.3	0.9	0.01
PVA-9 wt.% SSA		402.9	4.2	0.01
PVA-23 wt.% SSA		224.6	4.1	0.04
PVA-33 wt.% SSA		166.4	3.4	0.01
PVA-50 wt.% SSA		89.4	-	-
PVA-5 wt.% SSA	140	149.1	0.9	0.03
PVA-9 wt.% SSA		362.4	1.7	0.12
PVA-23 wt.% SSA		237.6	6.0	0.01
PVA-33 wt.% SSA		149.4	11.9	0.05
PVA-50 wt.% SSA		122.2	-	3.67
PVA-5 wt.% SSA	160	174.0	0.4	0.05
PVA-9 wt.% SSA		369.6	2.0	0.04
PVA-23 wt.% SSA		264.0	6.9	0.01
PVA-33 wt.% SSA		75.4	11.3	0.03
PVA-50 wt.% SSA		-	-	4.18

**Table 5:** Water vapor permeability coefficients of the PVA membranes.

Membrane	Water Activity [–]	*P* [Barrer] *	*P*·10^−4^ [g·mm·m^−2^·kPa^−1^·h^−1^]	*P*·10^−4^ [g·m·m^−2^·atm^−1^·d^−1^]	Reference
PVA-23 wt.% SSA 140 °C	0.24	17	4	9	This work
	0.41	18	4	10	This work
	0.59	2742	596	1451	This work
	0.68	9312	2022	4928	This work
	0.76	13759	2981	7264	This work
	0.85	32762	7113	17335	This work
	0.91	63865	13819	33675	This work
PVA-4-GA	0.60	-	1.5·10^−5^	-	[4]
PVOH*.CD	0.70	-	-	8.75	[6]
(PVA.CD)*	0.70	-	-	3.75	[6]

* 1 Barrer = 10^−10^ cm^3^_STP_·cm·cm^−2^·s^−1^·cm Hg^−1^.
